# Intraoperative Wound Irrigation in Orthopaedic Surgery: A Survey of Current Understanding and Practice Across the United States

**DOI:** 10.1016/j.artd.2025.101923

**Published:** 2025-12-16

**Authors:** Emma Woodmansey, Frank A. Buttacavoli, Aldo Riesgo, Christopher Bibbo, Nicholas Tedesco, David Rodriguez, Eric Lebby, Jonathan R. Danoff, Alberto V. Carli

**Affiliations:** a Clinical & Scientific Solutions, York, UK; b School of Medicine, Cardiff University, Cardiff, Wales, UK; c Department of Orthopedics, University of Texas Health San Antonio, San Antonio, TX, USA; d Department of Orthopedics, Baptist Health Orthopedic Institute, Miami, FL, USA; e Rubin Institute for Advanced Orthopaedics / International Center for Limb Lengthening, Sinai Hospital of Baltimore, Baltimore, MD, USA; f Department of Orthopedics, Samaritan Health Services, Corvallis, OR, USA; g Department of Orthopedics, University of Texas Health Houston, Houston, TX, USA; h Department of Orthopedics, Lehigh Valley Orthopedic Institute, Allentown, PA, USA; i Department of Orthopedics, Northwell Health Orthopedic Institute, Great Neck, NY, USA; j Department of Orthopedics, Hospital for Special Surgery, New York City, NY, USA

**Keywords:** Intraoperative wound irrigation, Antiseptics, Periprosthetic joint infection (PJI), Biofilm

## Abstract

**Background:**

Periprosthetic joint infections remain a serious complication following arthroplasty surgery, causing significant patient morbidity and economic burden to health-care systems. While surgical site infection (SSI) preventive measures have shown effectiveness, there remains a significant gap in literature regarding surgeon intraoperative practice, such as the use of intraoperative wound irrigation (IOWI). While studies highlight the potential in reducing SSIs, variability in clinical application and the lack of standardized, evidence-based guidelines necessitate a comprehensive understanding of current practices.

**Methods:**

A 46-question survey was developed following literature review and validation with high-volume primary and revision arthroplasty surgeons. Deployed via online clinician engagement platform, the survey queried challenges of SSI in relation to IOWI, current IOWI practice, the role of biofilm in periprosthetic joint infections, and ideal properties of irrigation solutions.

**Results:**

A total of 112 orthopaedic surgeons across the United States participated in the survey. Respondents indicated a high level of knowledge regarding the role of IOWI in SSI treatment and prevention. Key attributes of an ideal IOWI varied depending on procedural step (exposure, instrumentation, implantation, and closure) and procedure type (primary or revision). Variation in IOWI practice was evident in irrigant selection and decision rationale, with relatively lower alignment to contact time and residual antimicrobial activity.

**Conclusions:**

This survey highlights the perception that IOWI is an important part of routine SSI reduction measures and suggests variation in practice interventions and solution preference. Our findings support the necessity for a rigorous, evidence-based consensus via expert guidance to address the key surgical challenges to improve consistency of IOWI solution utilization.

## Introduction

Surgical site infections (SSIs), especially periprosthetic joint infections (PJIs), remain a significant challenge in total joint arthroplasty. [1,2] Patient risk factors and comorbidities for SSI’s, including high body mass index (>35 kg/m^2^), diabetes mellitus, smoking, and antimicrobial resistance, [[3], [4], [5], [6], [7]] among others, result in increased patient morbidity, prolonged hospital stays, and substantial health-care costs. [2,[8], [9], [10]]

Comprehensive prevention strategies encompass the entire perioperative process including preoperative, intraoperative, and postoperative interventions, with current guidelines and protocol development from academic and societal entities aiming to standardize evidence-based care and to decrease SSI rates globally. [[10], [11], [12]] The optimization of infection prevention protocols are especially critical given that the burden of revision arthroplasty is expected to increase in correlation to rising incidence of PJI. [13,14] This upward trend poses a significant clinical and economic challenge, as PJI is associated with increased patient mortality, possible limb amputation, poor functional outcomes, and substantial financial costs to the health-care ecosystem—with estimated annual hospital costs to be $1.85 billion by 2030 in the United States. [15,16] Ultimately, PJI may result in amputation, with a recent systematic review reporting rates of between 0.025% in primary total knee arthroplasty procedures and up to 5.1% in infected total knee arthroplasty revisions. [16] Several recent guidelines to prevent arthroplasty SSI’s highlight the role of intraoperative wound irrigation (IOWI) using antiseptics. [10,[17], [18], [19], [20], [21], [22]] Moreover IOWI has gained increasing attention for its role in reducing microbial contamination and biofilm formation. [[22], [23], [24], [25]] However, variations in irrigation practices, such as surgeon preference, irrigant formulations’ associated mechanism of action, and variations in implementation of IOWI makes comparing clinical outcome studies difficult. This raises concerns, especially as emerging research underscores the impact of biofilm on PJI [24,26] and the need for irrigation solutions that effectively balance antimicrobial efficacy with minimal cytotoxicity. [27] Furthermore, growing awareness of antimicrobial resistance and the need for antibiotic stewardship necessitates a reassessment of current SSI prevention strategies in relation to adjunctive local microbial management. [3,4,6,28]

Developing specific evidence-based recommendations on the proper implementation of IOWI solutions is anticipated to establish a standard of care. In order to develop recommendations, a baseline understanding of current surgeon practices and the ideal attributes of selecting an irrigation solution is necessary.

To the authors’ knowledge, surgeon variability and compliance to the variable recommendations for IOWI has not been evaluated. Due to the paucity of information related to real-world surgical use of IOWI, we aimed to capture the prevailing perceptions, common practice and knowledge gaps to inform future evidence-based recommendations to prevent SSI. We hypothesized that significant variability exists in both the use and perception of IOWI among orthopaedic surgeons, and the lack of standardized guidance contributes to inconsistent practices and outcomes in SSI prevention. To test these assumptions, the current study was designed with cross-sectional analysis of current practice trends and the adoption of such practices, by deploying a survey to high volume primary and revision arthroplasty surgeons across the United States. Specifically, the aims of the study were as follows: (1) define surgeons’ current perceptions of why SSIs occur and consequently their use of IOWIs in practice, (2) determine IOWI properties and function that surgeons consider as ideal, and (3) to identify surgeons’ perceptions for what future requirements would need to be met to successfully deploy guidelines for appropriate IOWI use.

## Material and methods

### Survey question development

A literature search was performed to inform survey question development. Three parallel searches were conducted in PubMed on September 18, 2024, with no limits or filters for search terminology ((*Antimicrobial*) AND (*irrigation*)) AND ("*Surgical Site Infection*")); OR (*SSI*)); AND (*wound*), ("*Periprosthetic Joint Infection*") AND (*Biofilm*), (*Antimicrobial resistance*) AND (*Orthopedic surgery*). Relevant articles were screened and reviewed, with a focus on applicability to orthopaedic surgery. From this review, 4 key sections were developed (current practice, intervention optimization, supporting research, and future research), comprising 46 data acquisition questions. These questions explored types of irrigation solutions used (saline or other) in the previous 2 years, the clinical rationale for selection in varying surgical procedures or stages, delivery method, solution volumes, and rinse steps. The questions also addressed biofilms as a clinical challenge and how this influenced clinician decision making on irrigation selection, and ideal properties of an irrigation solution, supported by both clinical and laboratory data. Survey questions were validated by in-depth discussion rationale, with 4 high-volume arthroplasty surgeons with experience comparable to the target respondent population. Following this process, the questions were edited for maximum data acquisition and implemented as the final survey questions (Supplementary Table 1).

### Survey implementation

Final survey questions were administered via an online independent physician engagement platform with access to global health-care professionals network (SERMO Inc., New York). Orthopaedic surgeons located in the United States were invited to complete the survey based on the following inclusion criteria: (1) board-certified orthopaedic surgeon; (2) greater than 200 joint replacement procedures annually; (3) a practice of utilizing IOWI solutions (saline or other); and (4) and a reported SSI rate greater than zero. Respondents failing to meet these criteria or not completing the survey were excluded. Participating surgeons were blinded to company brands or funding support of the survey. Additionally, the mention of specific proprietary IOWI solutions were generically referred. Demographic information included regional practice location, orthopaedic surgical specialty, and number of years in practice.

### Informed consent and ethical approvals

No informed consent or institutional review board approvals were required for this research.

### Survey data acquisition and analysis

Anonymous survey responses were downloaded in aggregate from the platform and specific quantifiable percentage data for each question was analyzed and summarized. No patient data or information was requested or discussed; therefore, was not deemed necessary for institutional review board or ethics committee approval.

## Results

**Figure 1:**
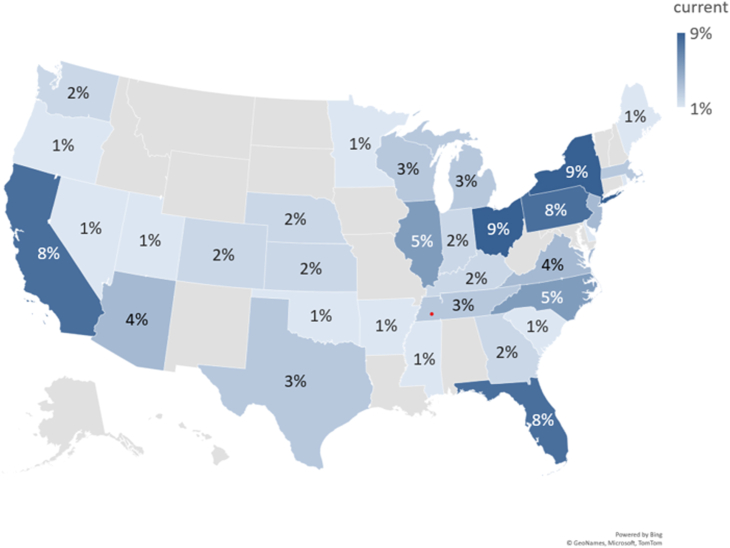
Representative distribution map of surgeon respondents by location across the United States (n = 112).

A total of 175 orthopaedic surgeons participated in the survey. Of the participating surgeons, 112 met the criteria to complete the survey, a response rate of 64%. All respondents practiced medicine in the United States with country wide representation (Fig. 1 and Supplementary Table 2).

**Figure 2:**
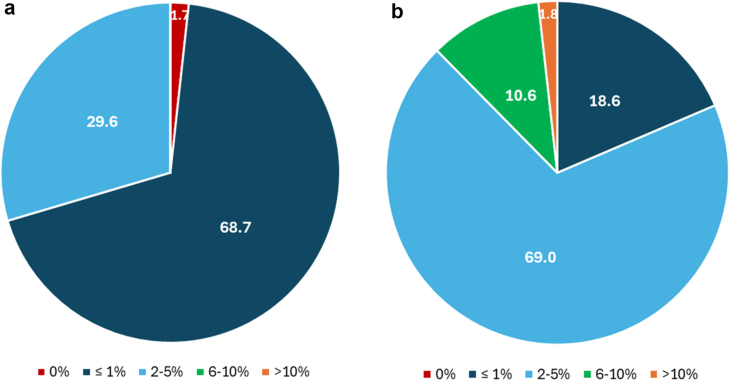
Self-reported SSI rate for (a) primary and (b) revision joint replacement (n = 112).

When queried on primary arthroplasty infection rates, 69.6% (n = 78) of respondents self-reported rates of <1%, while the remaining 30.4% (n = 34) of respondents self-reported between 2-5% (Fig. 2a). These rates were observed to be higher in revision cases, with 18.8% (n = 21) of respondents reporting an SSI rate of <1% and the majority (69.6%, n = 78) stating that 2-5% of revision surgeries suffered an SSI (Fig. 2b).

**Figure 3:**
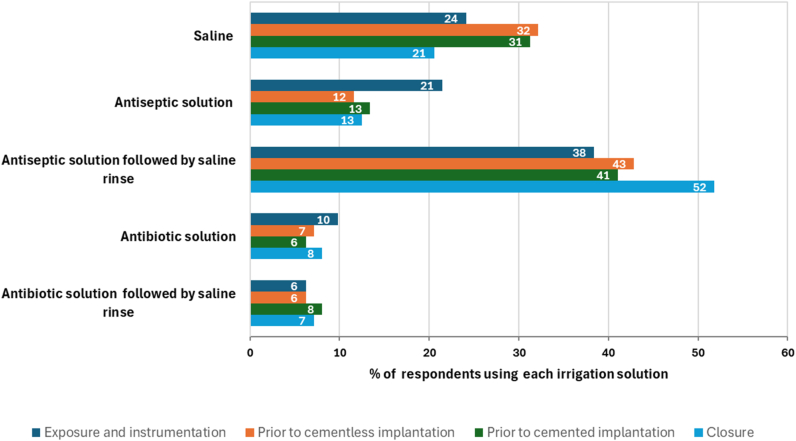
Type of irrigation solution utilized by procedure type or stage (n = 112).

When analyzing the most common type of irrigation solution utilized during the sequential operative procedural steps (exposure, instrumentation, prosthesis implantation, cementless or cemented, and closure), the greatest proportion of surgeons reported antiseptic irrigation solution followed by a saline rinse across all phases and procedures (Fig. 3). The greatest proportion (52%) of IOWI was reported to occur at closure. Saline alone was the next most frequently used irrigation solution across all procedural steps but particularly during implantation. A greater proportion of surgeons (21%) used a no-rinse antiseptic during exposure and instrumentation phase vs other stages (12-13%). Fewer surgeons used antibiotic solutions at any stage during the procedure.

**Figure 4:**
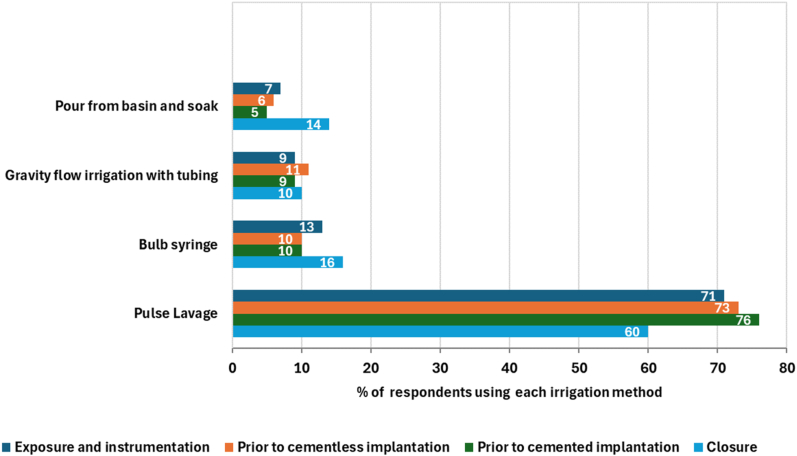
Delivery method for intraoperative irrigation solutions by procedure stage (n = 112).

With regard to IOWI delivery method, the majority of respondents preferred pulse lavage across all surgical procedure phases (Fig. 4). Soaks, gravity flow, and bulb syringe were used relatively infrequently across all stages.

**Figure 5:**
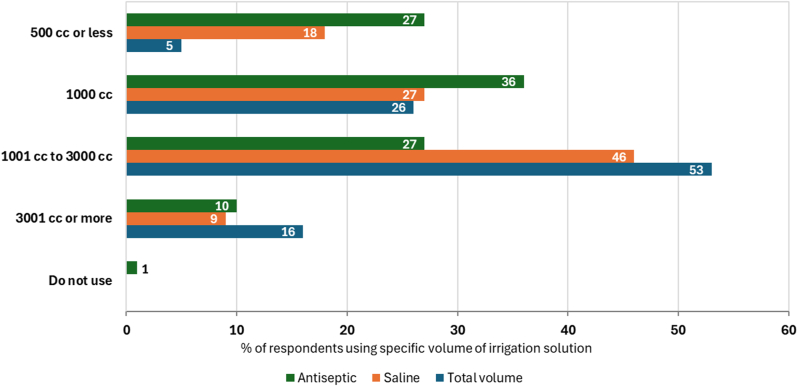
Volume of irrigation solutions, segmented by the total volume of all irrigation solutions, routinely used by surgeons across all procedure phases during a primary joint replacement (n = 112).

Nearly half of respondents reported use of large volumes (between 1000 mL to 3000 mL) of saline (Fig. 5). In comparison, lower quantities of antiseptic solutions were used, with more than 60% of respondents using 1000 mL or less of irrigant solution.

**Figure 6:**
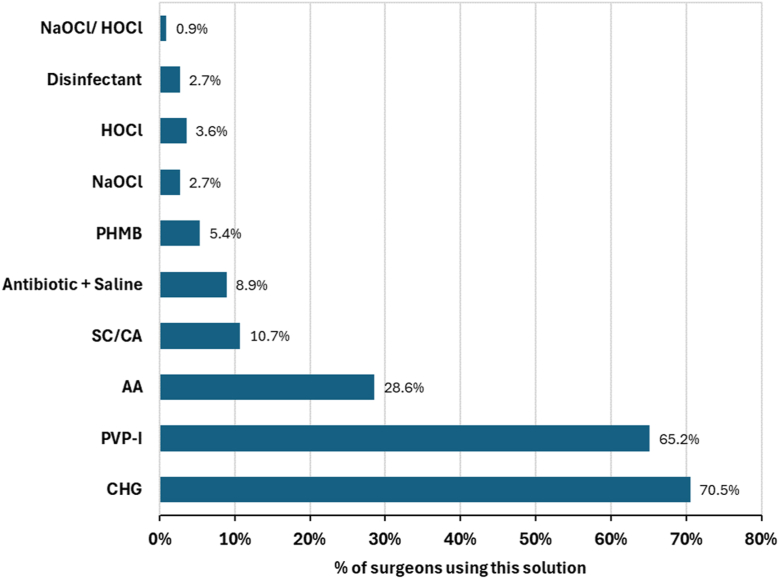
Most commonly used∗ irrigation solutions by generic ingredient (n = 112). AA, acetic acid; SC/CA, sodium citrate/citric acid; PHMB, polyhexamethylene biguanide; NaOCl, sodium hypochlorite; HOCl, hypochlorous acid. ∗Respondents were asked to list all irrigation solutions that they utilized within the past 24 months.

In addition to saline, irrigation solutions used by surgeons within the previous 24 months were reported (Fig. 6). Chlorhexidine gluconate (CHG) solution was the most commonly used irrigant solution by respondents (70.5%), followed by povidone iodine (PVP-I) (65.2%) and acetic acid (28.6%). Other irrigation solutions containing citric acid, polyhexamethylene biguanide, hydrogen peroxide, sodium hypochlorite or hypochlorous acid made up the majority of the remaining solutions utilized.

**Figure 7:**
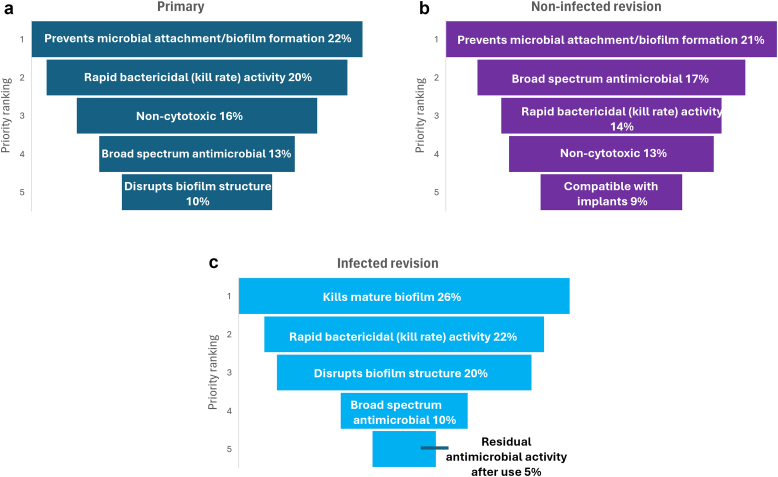
Priority ranking of the top 5 key attributes of an irrigation solution by (a) primary, (b) noninfected revision, and (c) infected revision (n = 112).

In evaluation, the ranking of key irrigant attributes of an ideal irrigation solution for primary- and noninfected revision-arthroplasty (Fig. 7a and b), respondents preferred prevention of microbial attachment as the main feature (22% and 21%). For infected cases (Fig. 7c), an irrigant solution possessing activity against mature biofilm was ranked to be of greater importance, in 26% of respondents. Rapid bactericidal activity was important across all surgery types (ranked number 2 in primary and infected procedures and number 3 in noninfected revisions). Broad spectrum antimicrobial activity was ranked in the top 5 of all procedure types but rated higher in primary (rank order 3) and noninfected revisions (rank 2) compared to infected revisions (ranked 4). Lack of cytotoxicity of an irrigation solution was noted in primary and noninfected cases (ranked 4, 16% and 13% respectively); however, was not ranked in top 5 for infected revision procedures. The ability of an irrigation solution to disrupt biofilms was reported as high ranking, particularly in infected cases, with 20% of surgeons rating this as the third most important feature. Additionally, 10% of respondents rated biofilm disruption as fifth priority for primary joint replacements. Residual antimicrobial activity received reduced priority for primary and noninfected procedures but did rank as the fifth most important attribute in infected cases.

**Figure 8:**
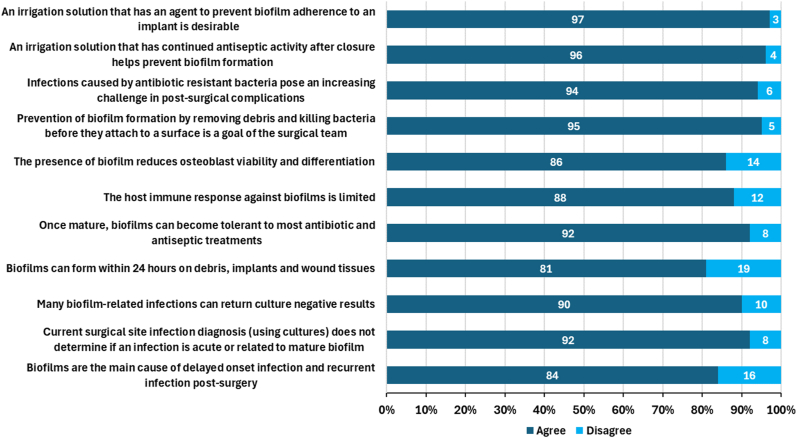
Respondent agreement with statements concerning the current challenges of biofilm in PJI (n = 112).

**Figure 9:**
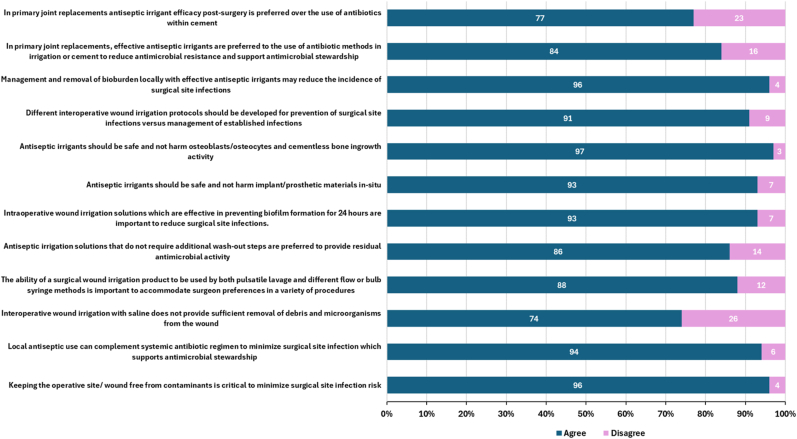
Respondent agreement with statements concerning intraoperative wound irrigation interventions (n = 112).

**Figure 10:**
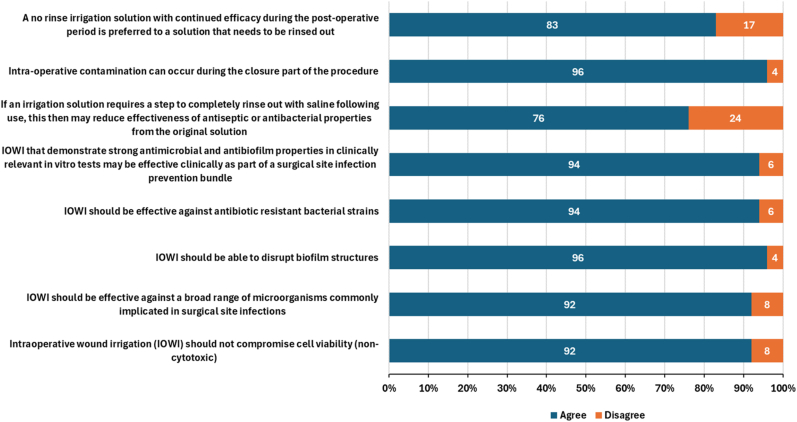
Respondent agreement with statements concerning in vitro expectations of intraoperative wound irrigation (n = 112).

Agreement with evidence-based statements relating to the challenge of biofilms in the setting of an active PJI, use of irrigation solutions interventions, and in vitro expectations of the ideal irrigation solution, are shown in Figure 8, Figure 9, Figure 10, respectively.

The majority of surgeons agreed that antiseptic solutions have a role in reducing contaminants and bioburden to minimize SSI, with the majority of respondents (96%) acknowledging that antiseptic use can complement systemic antibiotic use in this regard. Strong agreement was reported in properties such as efficacy against broad spectrum organisms (92%); including antibiotic resistant strains (94%); the ability to disrupt biofilms structures (96%); and the ideal antiseptic irrigation solution balanced with minimal cytotoxicity (92%). Furthermore, 94% of respondents align that the irrigation solutions that demonstrate strong antimicrobial and antibiofilm activity in clinically relevant in vitro tests may be effective as part of an SSI prevention bundle.

Furthermore, the clinical challenges posed by biofilms in total joint arthroplasty such as diagnosis difficulties and tolerance to treatment, align accordingly to agreement that irrigation solutions should prevent biofilm formation, even after closure and that solutions with residual activity may support this requirement. Respondents also agreed (91%) that different irrigation protocols should be used for prevention of infection vs treatment of established infection and that the requirement for a strong safety profile was important (antiseptics should not harm osteoblast or osteocyte viability (97%) or impact implant materials (93%)).

Less agreement was reported with the statement that that biofilms can form rapidly within 24 hours on debris, implants, and wound tissue and on biofilm impact on osteoblast viability and differentiation (86%) and the limited immune response against this phenotype (88%). In addition, 16% of respondents disagreed with the statement that biofilms are the main cause of delayed-onset infections and recurrent infections postsurgery.

Slight contradictions were noted in regard to no-rinse solutions and residual antiseptic activity; 86% respondents stated that they would prefer an antiseptic irrigation solution that does not require additional wash-out steps to aid residual antimicrobial activity, although 93% agreed that “irrigation solutions that can prevent biofilm formation for up to 24 hours are important in practice” and that “an antiseptic solution with residual activity may prevent biofilm formation” (96%). Moreover, 83% of respondents noted the positive impact an irrigation solution with continued antimicrobial efficacy may have in the postoperative period. However, despite 96% of respondents agreeing that contamination can occur at closure, only 76% agreed that washing the out the antimicrobial irrigation solution would reduce the ongoing antimicrobial efficacy.

## Discussion

The results of this United States survey present data from the clinical observations and identify perceptions, knowledge, and the practical experience of orthopaedic surgeons in addressing the prevention and management of SSI or PJIs. The data collected by this survey confirm the widespread use of intraoperative use of irrigation solutions during hip and knee arthroplasty surgery. These data also exhibit an agreement among the majority of respondents as to the core principles and rationale for performing IOWI, namely the removal of debris and contaminants, including microorganisms, from the wound in order to reduce the risk of developing an SSI.

Survey respondents report the use of various antiseptic irrigation solutions (54% to 65%) dependant on the stage of the procedure, with chlorhexidine and PVP-I–based irrigation solutions most frequently used (70.5% and 65.2% respectively). These results are in agreement with a similar survey of operative nurses and surgeons in Spain, that reported 41% using an antiseptic irrigation solution for wound irrigation. [29] Moreover, incorporating IOWI with antiseptic irrigation solutions has been shown across multiple clinical studies to assist in the reduction of developing SSIs. [19,20] Locally applied antiseptics can be used in much higher concentrations than systemic antibiotics, providing highly effective bactericidal properties to minimize contaminating bacteria. [25] A recent meta-analysis reported with a high level of certainty that IOWI with antiseptic irrigation solutions were associated with a significant reduction in SSIs when compared to no irrigation, even in clean-rated surgeries. [20] Similar findings were previously reported by the Cochrane group, with meta-analysis demonstrating there may be a lower incidence of SSI in patients treated with antibacterial irrigation compared to those treated with nonantibacterial irrigation. [19]

Generally, comparisons of SSI rates between different antiseptic irrigation solutions reported low consensus across studies on preferred agents. [19,30] This ambiguity, combined with surgeon preference and hospital protocols, provides explanation to the lack of clear evidence for specific agents and the wide range of various irrigation solutions reported to be used in this study. CHG and PVP-I–based solutions were reported to be the most frequently used by the respondents to this survey. For CHG especially, this is at odds with the limited clinical evidence supporting its use for IOWI, with the majority of clinical evidence supporting skin preparation only. [11] Furthermore, increasing concern for resistance and cross-resistance of antibiotics to CHG highlights the need for revisiting this current practice. [[31], [32], [33], [34]] Stronger randomized controlled trial level evidence does exist to support the choice of PVP-I solutions in IOWI, particularly in contaminated surgeries such as laparotomy procedures, [20,35] but its impact in cleaner wounds is less certain. [19,36] These perspectives highlight the need for further direct comparison clinical trials. Furthermore, the choice of solution may not align to the actual attributes deemed important across cases as reflected in some of the responses in this survey. For example, the most commonly used solutions reported in this data, CHG and PVP-I, both have rapid bactericidal activity (ranked number 2 or 3 in this survey) against planktonic organisms, [37] however, considering the top requirement across procedures to be effective against biofilms, data are mixed; both solutions have been shown to prevent biofilm formation in vitro, [38] although treatment of preformed biofilm is variable with CHG been shown to have some effect against biofilm phenotypes using an in vitro model of total joint infection, at high concentrations (2 and 4%) but lower concentrations aligned to less cytotoxicity (<1%) had minimal effect with significant regrowth of biofilm populations after 24 hours reincubation. [39] In contrast, PVP-I has shown good activity against staphylococcal biofilms in vitro across multiple surfaces including titanium and plastic. [37] However, use is cautioned over cytotoxicity concerns [40] (ranked number three in importance for primary procedures in this study) and systemic impacts on patients with thyroid conditions. [41]

Prioritization of key attributes that an irrigation solution should demonstrate depends on the issue presented to the surgeon. For example, a clean operative site in primary joint replacement has differing priorities compared to an infected wound in revision surgery, as reflected in this survey. Moreover, the support to reducing infection risk and optimizing recovery is of upmost importance. For these reasons, use of irrigation solutions that are noncytotoxic to tissue and bone cells is critical; [27,40,42] both PVP-I and hydrogen peroxide have been shown to reduce migration and proliferation of fibroblasts in vitro in a dose-dependent manner. [40] Chlorhexidine and bacitracin have demonstrated an impact on osteoblast morphology and proliferation in vitro. [42] This balance of risk-to-benefit consideration was apparent in responses, with respondents indicating that cytotoxicity was deemed less important when the contamination risk increased in revision and infected revision cases, presumably due to the increased priority to remove microorganisms.

Equally important, evidence has demonstrated that microbial presence and biofilm formation negatively affects osteoblast viability and function. In particular, *Staphylococcus aureus* biofilms are shown to reduce differentiation and viability [43] with recent studies highlighting dysregulation of progenitor cells in vitro and significant reduction to bone thickness in vivo. [44] Understanding of how biofilm can affect osteoblast viability and activity was less evident in this study, where relatively less agreement (86%) was reported for this statement. This may be a result of lack of point of care diagnostics that specifically differentiate biofilm infection from planktonic (acute) infection and thus, is not routinely monitored in infected or revision cases. [45] Furthermore, additional education, specifically regarding biofilms, may be beneficial to build understanding as to how different biofilm phenotypes require alternative management to traditional treatment of infections.

Many late onset and recurrent infections are biofilm associated. [24,26,45,46] Once established bacterial biofilms are a key source of PJIs and are often resistant to treatment by antibiotics alone. [24,47] For this reason, the prevention of biofilm in any surgical site is an important step to prevent a PJI. [24] Our study data clearly demonstrate surgeon opinion as to the general concept of preventing microbial attachment to implants and resultant biofilm formation as being the top priority in primary joint replacements, whereas disruption of biofilm becomes increasingly important in revision cases.

Rapid antimicrobial activity is important to minimize risk of developing resistance and to effectively kill antibiotic resistant organisms. [48] Respondents in this survey agreed with this parameter and furthermore supported the role of antiseptics to complement systemic antibiotic use to minimize SSI; thus, supporting antimicrobial stewardship, something long advocated in the infectious disease community. [25,49] An effective and rapid bacterial kill rate with irrigation solutions was deemed important across both primary and revision procedures by the respondents in this study. While there is no clear evidence on the ideal soak or lavage irrigation duration, many opinions promote a duration of up to 3 minutes. [50] To balance effectiveness vs potential cytotoxicity in solutions that require a rinse step, it is critical that antimicrobial activity is imparted within the selected duration to have a meaningful impact on reducing contamination. Additionally, evaluating irrigations solutions under clinically relevant conditions is crucial, as recent research indicates that some antiseptic cleansers exhibit limited efficacy with brief dwell times. [51]

Despite agreed challenges of contamination during the closure step, a lower agreement was observed in this survey regarding rinse steps following the use of antiseptic irrigation solutions. Theoretically, solutions that require no saline rinse after use may practically reduce operation duration combined with providing ongoing antimicrobial activity in the operative site helping to reduce microbial contamination if it occurs. Such residual activity was not high priority in primary or noninfected procedures based on the results of this survey; however, was more important (ranked number 5) in infected cases. These results highlight an area for further guidance to bridge this knowledge gap linking to areas such as prevention of microbial attachment and antimicrobial contact times.

This survey has several limitations. As the survey was limited to respondents practicing in the United States, generalizability of these findings may be limited internationally. Also, antiseptic agents and practice patterns may differ outside the United States and so responses may be biased. Further, the digital circulation of the survey to surgeons limited the ability to verify number of procedures performed annually. In addition, there may be bias toward surgeons who are more likely to respond to surveys and so it may not reflect the practice patterns of surgeons who are not academically inclined to have interest in this area.

Overall, the results of this survey are in alignment with the current evidence supporting IOWI using antiseptic irrigation solutions. Given the shortage of comparative evidence identifying an ideal antiseptic agent, expert opinion and experience must be relied on to guide best practice. Further comparative clinical studies should be performed using solutions that show promising outcomes in preclinical testing to aid differentiation between agents. Moreover, the development of evidence-based consensus and expert guidance would support enhanced consistency of use in practice, improve quality of care delivery, and help optimize IOWI use.

## Conclusions

This survey of practicing of high-volume, United States–based, adult reconstruction orthopaedic surgeons highlight the perception that IOWI is an important part of routine SSI reduction measures. In addition, the results suggest variation in practice patterns, with preference on different solutions used. The most important attributes of an ideal irrigation solution were dependent on type of surgical procedure with prevention of biofilm formation and rapid speed of kill ranked highest in primary procedures, whereas ability to disrupt and kill biofilms was much more imperative in infected cases. This survey study highlights that the types of antiseptic solutions used do not necessarily match the key attributes sought in an ideal antiseptic irrigation solution. These findings suggest the necessity for wider education and standardized practice guidance, which may address key surgical challenges and, ultimately, enable improved consistency in practice.

## Funding

This work was sponsored by Sanara MedTech Inc. Fort Worth, Texas.

## Conflicts of interest

Emma Woodmansey is a paid consultant for Sanara MedTech Inc and Accel Heal and owns stock or stock options in Smith &Nephew.

Frank Buttacavoli is on the speakers' bureau or receives paid presentations for and is a paid consultant for Zimmer Biomet, Sanara MedTech, Solventum, Heraus, and Medtronic and is a board member or attends committee appointments for the AAOS Hip Knee Evaluation Committee.

Aldo Riesgo is a paid consultant for Zimmer Biomet, Stryker, and Sanara MedTech; receives research support as a principal investigator from Cleu Diagnostics Inc.

Christopher Bibbo is on the speakers' bureau or receives paid presentations for and is a paid consultant for Sanara MedTech.

Nicholas Tedesco is on the speakers' bureau or receives paid presentations for Sanara MedTech; owns stock or stock options in ROMTech and Doctorpedia; and is a deputy editor at the oncology section for JAOAO; is a president at the MORI Board, MSTS Committee Chair, and is a member of the AAOS EAEC Committee.

David Rodriguez receives royalties and research support from Zimmer Biomet and is a paid consultant for Zimmer Biomet and Sanara MedTech.

Eric Lebby is a paid consultant for Sanara MedTech.

Jonathan R. Danoff is a paid consultant for Stryker, Surgical Specialties, and Sanara MedTech; serves as an editor for Arthroplasty Today; and is a board member or attends committee appointments for AAHKS.

Alberto V. Carli is a paid consultant for Hereaus Medical, Zimmer Biomet, and Sanara MedTech.

For full disclosure statements refer to https://doi.org/10.1016/j.artd.2025.101923.

## CRediT authorship contribution statement

**Emma Woodmansey:** Writing – review & editing, Writing – original draft, Visualization, Validation, Project administration, Methodology, Funding acquisition, Formal analysis, Data curation, Conceptualization. **Frank A. Buttacavoli:** Writing – review & editing, Validation. **Aldo Riesgo:** Writing – review & editing, Validation. **Christopher Bibbo:** Writing – review & editing, Validation. **Nicholas Tedesco:** Writing – review & editing, Validation. **David Rodriguez:** Writing – review & editing, Validation. **Eric Lebby:** Writing – review & editing, Visualization. **Jonathan R. Danoff:** Writing – review & editing, Visualization, Validation, Supervision. **Alberto V. Carli:** Writing – review & editing, Visualization, Validation, Supervision.

## Figures and Tables

**Supplementary Table 2:** Survey respondent demographics.

Surgeon parameter	Number (percentage)
Number of surgeons screened	175
Number of respondents meeting the qualifying criteria	112
Number of surgeons performing >200 orthopaedic procedures/ year	112 (100)
Primary orthopaedics specialty	
Joint replacement	98 (87.5)
Sports medicine	8 (7.1)
Trauma	6 (5.4)
Years in practice	
>25	10 (8.9)
16-25	26 (23.2)
6-15	64 (57.1)
≤5	12 (10.7)

## Appendix A Supplementary data

Conflict of Interest Statement for Bibbo

Conflict of Interest Statement for Buttacavoli

Conflict of Interest Statement for Carli

Conflict of Interest Statement for Danoff

Conflict of Interest Statement for Lebby

Conflict of Interest Statement for Riesgo

Conflict of Interest Statement for Rodriguez

Conflict of Interest Statement for Tedesco

Conflict of Interest Statement for Woodmansey

## References

[1] Purcell R.L., Parks N.L., Cody J.P., Hamilton W.G. (2018). Comparison of wound complications and deep infections with direct anterior and posterior approaches in Obese hip arthroplasty patients. J Arthroplasty.

[2] Statz J.M., Duethman N.C., Trousdale R.T., Taunton M.J. (2019). Outcome of direct anterior total hip arthroplasty complicated by superficial wound dehiscence requiring irrigation and debridement. J Arthroplasty.

[3] Salisbury A.-M., Woo K., Sarkar S., Schultz G., Malone M., Mayer D.O. (2018). Tolerance of biofilms to antimicrobials and significance to antibiotic resistance in wounds. Surg Technol Int.

[4] Birgand G., Dhar P., Holmes A. (2023). The threat of antimicrobial resistance in surgical care: the surgeon’s role and ownership of antimicrobial stewardship. Br J Surg.

[5] Foschi D., Yakushkina A., Cammarata F., Lamperti G., Colombo F., Rimoldi S. (2022). Surgical site infections caused by multi-drug resistant organisms: a case–control study in general surgery. Updates Surg.

[6] Nagata K., Yamada K., Shinozaki T., Miyazaki T., Tokimura F., Tajiri Y. (2022). Effect of antimicrobial prophylaxis duration on health care–associated infections after clean orthopedic surgery. JAMA Netw Open.

[7] Whelan L., Leal J., Barkema H.W., Leslie M., McClure J.-A., Zhang K. (2023). Baseline prevalence of antimicrobial resistance in patients who develop a surgical site infection in hip and knee replacements: a brief report. Am J Infect Control.

[8] Florschutz A.V., Fagan R.P., Matar W.Y., Sawyer R.G., Berrios-Torres S.I. (2015). Surgical site infection risk factors and risk stratification. J Am Acad Orthop Surg.

[9] Korol E., Johnston K., Waser N., Sifakis F., Jafri H.S., Lo M. (2013). A systematic review of risk factors associated with surgical site infections among surgical patients. PLoS One.

[10] Calderwood M.S., Anderson D.J., Bratzler D.W., Dellinger E.P., Garcia-Houchins S., Maragakis L.L. (2023). Strategies to prevent surgical site infections in acute-care hospitals: 2022 update. Infect Control Hosp Epidemiol.

[11] Berríos-Torres S.I., Umscheid C.A., Bratzler D.W., Leas B., Stone E.C., Kelz R.R. (2017). Centers for Disease Control and Prevention Guideline for the prevention of surgical site infection, 2017. JAMA Surg.

[12] World Health Organization (2018).

[13] Schwartz A.M., Farley K.X., Guild G.N., Bradbury T.L. (2020). Projections and epidemiology of revision hip and knee arthroplasty in the United States to 2030. J Arthroplasty.

[14] Parvizi J., Tan T.L., Goswami K., Higuera C., Della Valle C., Chen A.F. (2018). The 2018 definition of periprosthetic hip and knee infection: an evidence-based and validated criteria. J Arthroplasty.

[15] Premkumar A., Kolin D.A., Farley K.X., Wilson J.M., McLawhorn A.S., Cross M.B. (2021). Projected economic burden of periprosthetic joint infection of the hip and knee in the United States. J Arthroplasty.

[16] Mousavian A., Sabzevari S., Ghiasi S., Shahpari O., Razi A., Ebrahimpour A. (2021). Amputation as a complication after total knee replacement, is it a real concern to be discussed?: a systematic review. Arch Bone Jt Surg.

[17] Bath M., Davies J., Suresh R., Machesney M. (2022). Surgical site infections: a scoping review on current intraoperative prevention measures. Ann The R Coll Surg Engl.

[18] Thom H., Norman G., Welton N.J., Crosbie E.J., Blazeby J., Dumville J.C. (2021). Intra-Cavity lavage and wound irrigation for prevention of surgical site infection: systematic review and network meta-analysis. Surg Infect (Larchmt).

[19] Norman G., Atkinson R.A., Smith T.A., Rowlands C., Rithalia A.D., Crosbie E.J. (2017). Intracavity lavage and wound irrigation for prevention of surgical site infection. Cochrane Database Syst Rev.

[20] Groenen H., Bontekoning N., Jalalzadeh H., Buis D.R., Dreissen Y.E.M., Goosen J.H.M. (2024). Incisional wound irrigation for the prevention of surgical site infection. JAMA Surg.

[21] O’Neal P.B., Itani K.M.F. (2016). Antimicrobial formulation and delivery in the prevention of surgical site infection. Surg Infect (Larchmt).

[22] Edmiston C.E., Leaper D.J. (2016). Intra-Operative surgical irrigation of the surgical incision: what does the future Hold—Saline, antibiotic agents, or antiseptic agents?. Surg Infect (Larchmt).

[23] Edmiston C.E., Leaper D.J. (2022). Prevention of orthopedic prosthetic infections using evidence-based surgical site infection care bundles: a narrative review. Surg Infect (Larchmt).

[24] Edmiston C.E., McBain A.J., Kiernan M., Leaper D.J. (2016). A narrative review of microbial biofilm in postoperative surgical site infections: clinical presentation and treatment. J Wound Care.

[25] Edmiston C.E., Spencer M., Leaper D. (2018). Antiseptic irrigation as an effective interventional strategy for reducing the risk of surgical site infections. Surg Infect (Larchmt).

[26] Song Z., Borgwardt L., Hoiby N., Wu H., Sorenson T.S., Borgwardt A. (2013). Prosthesis infections after orthopedic joint replacement: the possible role of bacterial biofilms. Orthop Rev.

[27] Geng R.S.Q., Sibbald R.G., Slomovic J., Toksarka O., Schultz G. (2025). Therapeutic indices of topical antiseptics in wound care: a systematic review. Adv Skin Wound Care.

[28] Edmiston C.E., Leaper D., Spencer M., Truitt K., Litz Fauerbach L., Graham D. (2017). Considering a new domain for antimicrobial stewardship: topical antibiotics in the open surgical wound. Am J Infect Control.

[29] Badia J.M., Rubio-Pérez I., López-Menéndez J., Diez C., Al-Raies Bolaños B., Ocaña-Guaita J. (2020). The persistent breach between evidence and practice in the prevention of surgical site infection. Qualitative study. Int J Surg.

[30] Caid M., Valk J., Danoff J. (2022). Irrigation solutions in total joint arthroplasty. Spartan Med Res J.

[31] Sultan A.M., Ahmed M.A. (2022). Distribution of chlorhexidine resistance genes among Staphylococcus aureus clinical isolates: the challenge of antiseptic resistance. Germs.

[32] Kõljalg S., Naaber P., Mikelsaar M. (2002). Antibiotic resistance as an indicator of bacterial chlorhexidine susceptibility. J Hosp Infect.

[33] Kampf G. (2016). Acquired resistance to chlorhexidine – is it time to establish an ‘antiseptic stewardship’ initiative?. J Hosp Infect.

[34] Abbood H.M., Hijazi K., Gould I.M. (2023). Chlorhexidine resistance or Cross-Resistance, that is the question. Antibiotics.

[35] Mueller T., Dimpel R., Kehl V., Friess H., Reim D. (2023). Surgical site infection prevention in abdominal surgery: is intraoperative wound irrigation with antiseptics effective? Protocol for a systematic review and meta-analysis. BMJ Open.

[36] Simon S.J., Grant A.R., Travers H.I., Smith E.L., Hollenbeck B.L. (2025). Povidone-Iodine versus saline irrigation on reduction of surgical site infections in total hip and knee arthroplasty: a retrospective, propensity-matched cohort. Surg Infect (Larchmt).

[37] Premkumar A., Nishtala S.N., Nguyen J.T., Bostrom M.P.G., Carli A.V. (2021). The AAHKS best podium presentation research award: comparing the efficacy of irrigation solutions on staphylococcal biofilm formed on arthroplasty surfaces. J Arthroplasty.

[38] Coles V.E., Puri L., Bhandari M., Wood T.J., Burrows L.L. (2024). The effects of chlorhexidine, povidone-iodine and vancomycin on growth and biofilms of pathogens that cause prosthetic joint infections: an in-vitro model. J Hosp Infect.

[39] Smith D.C., Maiman R., Schwechter E.M., Kim S.J., Hirsh D.M. (2015). Optimal irrigation and debridement of infected total joint implants with chlorhexidine gluconate. J Arthroplasty.

[40] Thomas G.W., Rael L.T., Bar-Or R., Shimonkevitz R., Mains C.W., Slone D.S. (2009). Mechanisms of delayed wound healing by commonly used antiseptics. J Trauma Inj Infect Crit Care.

[41] Nobukuni K., Hayakawa N., Namba R., Ihara Y., Sato K., Takada H. (1997). The influence of long-term treatment with povidone-iodine on thyroid function. Dermatology.

[42] Markel J.F., Bou-Akl T., Dietz P., Afsari A.M. (2021). The effect of different irrigation solutions on the cytotoxicity and recovery potential of human osteoblast cells in vitro. Arthroplast Today.

[43] Sanchez C.J., Ward C.L., Romano D.R., Hurtgen B.J., Hardy S.K., Woodbury R.L. (2013). Staphylococcus aureus biofilms decrease osteoblast viability, inhibits osteogenic differentiation, and increases bone resorption in vitro. BMC Musculoskelet Disord.

[44] Mouton W., Josse J., Jacqueline C., Abad L., Trouillet-Assant S., Caillon J. (2021). Staphylococcus aureus internalization impairs osteoblastic activity and early differentiation process. Sci Rep.

[45] Staats A., Li D., Sullivan A.C., Stoodley P. (2021). Biofilm formation in periprosthetic joint infections. Ann Jt.

[46] Costerton J.W., Stewart P.S., Greenberg E.P. (1999). Bacterial biofilms: a common cause of persistent infections. Science.

[47] McConoughey S.J., Howlin R., Granger J.F., Manring M.M., Calhoun J.H., Shirtliff M. (2014). Biofilms in periprosthetic orthopedic infections. Future Microbiol.

[48] Chopra I. (2007). The increasing use of silver-based products as antimicrobial agents: a useful development or a cause for concern?. J Antimicrob Chemother.

[49] Roberts C.D., Leaper D.J., Assadian O. (2017). The role of topical antiseptic agents within antimicrobial stewardship strategies for prevention and treatment of surgical site and chronic open wound infection. Adv Wound Care (New Rochelle).

[50] Charoenwisetsin S., Jiranantarat V., Hirunyachoke P., Udomkiat P. (2024). Effect of intraoperative cold solution irrigation to reduce postoperative pain in knee osteoarthritis patients who underwent unilateral primary total knee arthroplasty: a double-blinded randomized controlled trial. BMC Musculoskelet Disord.

[51] Johani K., Malone M., Jensen S.O., Dickson H.G., Gosbell I.B., Hu H. (2018). Evaluation of short exposure times of antimicrobial wound solutions against microbial biofilms: from in vitro to in vivo. J Antimicrob Chemother.

