# Supplementary material for: Intraoperative Wound Irrigation in Orthopaedic Surgery: A Survey of Current Understanding and Practice Across the United States
Source: Arthroplast Today. 2025 Dec 16;37:101923. doi: 10.1016/j.artd.2025.101923 (PMC12768874; doi:10.1016/j.artd.2025.101923)
Supplement: Conflict of Interest Statement for Tedesco [file mmc8.pdf]

ARTD-D-25-00305R1
Title: Intraoperative wound irrigation in orthopedic surgery: A survey of current understanding and practice across the United States

CONFLICT OF INTEREST STATEMENT

American Association of Hip and Knee Surgeons

(Adopted from the American Academy of Orthopaedic Surgeons disclosure statement)

The following form **must be filled out completely and submitted by each author (example, 6 authors, 6 forms). All items require a response. If there is no relevant disclosure for a given item, enter "*None.*"**

Manuscript Title

1. Royalties from a company or supplier (The following conflicts were disclosed)

2. Speakers bureau/paid presentations for a company or supplier (The following conflicts were disclosed)
   SANARA MEDTECH

3A. Paid employee for a company or supplier (The following conflicts were disclosed)

3B. Paid consultant for a company or supplier (The following conflicts were disclosed)

3C. Unpaid consultants for a company or supplier (The following conflicts were disclosed)

4. Stock or stock options in a company or supplier (The following conflicts were disclosed)
   ROM TECH, DOCTORPEDIA

5. Research support from a company or supplier as a Principal Investigator (The following conflicts were disclosed)

6. Other financial or material support from a company or supplier (The following conflicts were disclosed)

7. Royalties, financial or material support from publishers (The following conflicts were disclosed)

8. Medical/Orthopaedic publications editorial/governing board (The following conflicts were disclosed)
   DEPUTY EDITOR, ONCOLOGY SECTION, JAOAO

9. Board member/committee appointments for a society (The following conflicts were disclosed)
   MORI BOARD PRESIDENT, MSTS GEBM COMMITTEE CHAIR, AAOS EAEC COMMITTEE MEMBER

Each author must sign AND print or type his/her name, date and submit a separate form

In addition, one BLINDED Conflict of Interest form (no author names used) should be submitted per manuscript with all author disclosures.

NICHOLAS TEDESCO	[signature]	2/22/2025
Author Name (Print or Type)	Author Signature	Date
