# Supplementary material for: Intraoperative Wound Irrigation in Orthopaedic Surgery: A Survey of Current Understanding and Practice Across the United States
Source: Arthroplast Today. 2025 Dec 16;37:101923. doi: 10.1016/j.artd.2025.101923 (PMC12768874; doi:10.1016/j.artd.2025.101923)
Supplement: Conflict of Interest Statement for Woodmansey [file mmc9.pdf]

ARTD-D-25-00305R1
Title: Intraoperative wound irrigation in orthopedic surgery: A survey of current understanding and practice across the United States

CONFLICT OF INTEREST STATEMENT

American Association of Hip and Knee Surgeons

(Adopted from the American Academy of Orthopaedic Surgeons disclosure statement)

The following form **must be filled out completely and submitted by each author (example, 6 authors, 6 forms). All items require a response. If there is no relevant disclosure for a given item, enter "*None.*"**

Manuscript Title

1. Royalties from a company or supplier (The following conflicts were disclosed)

   NONE

2. Speakers bureau/paid presentations for a company or supplier (The following conflicts were disclosed)

   NONE

3A. Paid employee for a company or supplier (The following conflicts were disclosed)

   N/A. NONE

3B. Paid consultant for a company or supplier (The following conflicts were disclosed)

   PAID CONSULTANT FOR SANARA ~~[illegible]~~ MEDTECH INC. US.
   ACCEL-HEAL LTD, UK

3C. Unpaid consultants for a company or supplier (The following conflicts were disclosed)

   NONE

4. Stock or stock options in a company or supplier (The following conflicts were disclosed)

   SMITH & NEPHEW

5. Research support from a company or supplier as a Principal Investigator (The following conflicts were disclosed)

   NONE

6. Other financial or material support from a company or supplier (The following conflicts were disclosed)

   NONE

7. Royalties, financial or material support from publishers (The following conflicts were disclosed)

   NONE

8. Medical/Orthopaedic publications editorial/governing board (The following conflicts were disclosed)

   NONE

9. Board member/committee appointments for a society (The following conflicts were disclosed)

   NONE

Each author must sign AND print or type his/her name, date and submit a separate form

In addition, one BLINDED Conflict of Interest form (no author names used) should be submitted per manuscript with all author disclosures.

EMMA WOODMANSEY	[signature]	22nd February 2025
Author Name (Print or Type)	Author Signature	Date
