# Supplementary material for: Intraoperative Wound Irrigation in Orthopaedic Surgery: A Survey of Current Understanding and Practice Across the United States
Source: Arthroplast Today. 2025 Dec 16;37:101923. doi: 10.1016/j.artd.2025.101923 (PMC12768874; doi:10.1016/j.artd.2025.101923)
Supplement: Supplementary Table 1 [file mmc10.docx]

**Supplementary table 1.** Survey questions

**Screener questions**

***Screener questions may limit the number of guaranteed completes and will be displayed to respondents on a separate page.**

**S1) Are you a board certified orthopedic surgeon?**

Screening criteria: Screen out immediately if S1 = No

*Choose one answer*

○ Yes

○ No

**S2) Do you perform greater than 200 hip and knee joint replacement procedures per year?**

Screening criteria: Screen out immediately if S2 = No

*Choose one answer*

○ Yes

○ No

**S3) Roughly what is the rate of Surgical Site Infections (SSI) in your primary hip and knee replacements:**

Screening criteria: Screen out immediately if S3 = 0%

*Choose one answer*

○ 0%

○ ≤ 1%

○ 2-5%

○ 6-10%

○ > 10%

**S4) Roughly what is the rate of SSI in your revision hip and knee replacements:**

Screening criteria: Screen out immediately if S4 = 0%

*Choose one answer*

○ 0%

○ ≤ 1%

○ 2-5%

○ 6-10%

○ > 10%

**S5) Do you routinely use Intraoperative wound irrigation?**

Screening criteria: Screen out immediately if S5 = No

*Choose one answer*

○ Yes

○ No

**S6) What is your primary specialty in orthopedics?**

*Choose one answer* | Answers will be randomized

○ Joint replacement

○ Sports Medicine

○ Trauma

○ Musculoskeletal Oncology

**S7) What are the number of years you have been in practice?**

*Choose one answer*

○ Under 5

○ 6 to 15

○ 16 to 25

○ Over 25

**Survey questions**

**Q1) What is your most commonly used irrigation fluid during the exposure and instrumentation part of the procedure?**

*Choose one answer*

○ Saline

○ Antiseptic solution (Examples: Iodine, Chlorhexidine, PHMB, etc . . .)

○ Antiseptic solution followed by saline rinse

○ Antibiotic solution (Examples: Kanamycin, Bacitracin, Neomycin, etc . . .)

○ Antibiotic solution followed by saline rinse

**Q2) What method of irrigation/lavage do you predominantly use during the exposure and instrumentation part of the procedure?**

*Choose one answer* | Answers will be randomized

○ Pour from basin and soak

○ Gravity flow irrigation with tubing

○ Bulb syringe

○ Pulse lavage

**Q3) What is your most commonly used irrigation fluid prior to cementless component implantation?**

*Choose one answer*

○ Saline

○ Antiseptic solution

○ Antiseptic solution followed by saline rinse

○ Antibiotic solution

○ Antibiotic solution followed by saline rinse

**Q4) What method of irrigation/lavage do you predominantly use prior to cementless component implantation?**

*Choose one answer* | Answers will be randomized

○ Pour from basin and soak

○ Gravity flow irrigation with tubing

○ Bulb syringe

○ Pulse lavage

**Q5) What is your most commonly used irrigation fluid prior to cemented component implantation?**

*Choose one answer*

○ Saline

○ Antiseptic solution

○ Antiseptic solution followed by saline rinse

○ Antibiotic solution

○ Antibiotic solution followed by saline rinse

**Q6) What method of irrigation/lavage do you predominantly use prior to cemented component implantation?**

*Choose one answer* | Answers will be randomized

○ Pour from basin and soak

○ Gravity flow irrigation with tubing

○ Bulb syringe

○ Pulse lavage

**Q7) What is your most commonly used final irrigation fluid during closure?**

*Choose one answer*

○ Saline

○ Antiseptic solution

○ Antiseptic solution followed by saline rinse

○ Antibiotic solution

○ Antibiotic solution followed by saline rinse

**Q8) What method of irrigation/lavage do you predominantly use during closure?**

*Choose one answer* | Answers will be randomized

○ Pour from basin and soak

○ Gravity flow irrigation with tubing

○ Bulb syringe

○ Pulse lavage

**Q9) What is the total volume of irrigation you use for a routine joint replacement procedure? (This includes exposure, implantation and closure)**

*Choose one answer*

○ 500 cc or less

○ 1000 cc

○ 1001 cc to 3000 cc

○ 3001 cc or more

**Q10) Of the total volume of irrigation you use for a routine primary joint replacement, how much is saline?**

*Choose one answer*

○ 500 cc or less

○ 1000 cc

○ 1001 cc to 3000 cc

○ 3001 cc or more

**Q11) When using an antiseptic or antibacterial irrigation solution, what is the ideal total volume of irrigation you desire for a routine joint replacement procedure?**

*Choose one answer*

○ 500 cc or less

○ 1000 cc

○ 1001 cc to 3000 cc

○ 3001 cc or more

○ Do not use

**Q12) In addition to saline, please list up to five of the most common brand name irrigation solutions that you have used in the past two years. Please also indicate if you are still currently using the solution.**Example:
1. Brand A - yes
2. Brand B - no
3. Brand C - yes

*Enter at least 1 answer to continue*

**Q13) What are the five most important properties you require in an irrigation fluid for a *primary* total joint?**Please rank the top five with 1 (top) being the most important.

*Drag answer options to adjacent box, and rank in orderDrag answer options to below box, and rank in order
Rank 5 items to continue* | Answers will be randomized

↕ Non-cytotoxic

↕ Prevents microbial attachment/biofilm formation

↕ Broad spectrum antimicrobial

↕ Rapid bactericidal (kill rate) activity

↕ Disrupts biofilm structure

↕ Kills mature biofilm

↕ Compatible with implants

↕ Residual antimicrobial activity after use

↕ No rinse required

**Q14) What are the five most important properties you require in an irrigation fluid for a non-infected *revision* total joint?**Please rank the top five with 1 (top) being the most important.

*Drag answer options to adjacent box, and rank in orderDrag answer options to below box, and rank in order
Rank 5 items to continue* | Answers will be randomized

↕ Non-cytotoxic

↕ Prevents microbial attachment/biofilm formation

↕ Broad spectrum antimicrobial

↕ Rapid bactericidal (kill rate) activity

↕ Disrupts biofilm structure

↕ Kills mature biofilm

↕ Compatible with implants

↕ Residual antimicrobial activity after use

↕ No rinse required

**Q15) What are the five most important properties you require in an irrigation fluid for *infected* total joint procedures?**Please rank the top five with 1 (top) being the most important.

*Drag answer options to adjacent box, and rank in orderDrag answer options to below box, and rank in order
Rank 5 items to continue* | Answers will be randomized

↕ Non-cytotoxic

↕ Prevents microbial attachment/biofilm formation

↕ Broad spectrum antimicrobial

↕ Rapid bactericidal (kill rate) activity

↕ Disrupts biofilm structure

↕ Kills mature biofilm

↕ Compatible with implants

↕ Residual antimicrobial activity after use

↕ No rinse required

**Q16) Do you agree or disagree with the following statements regarding c*urrent* Challenges of Biofilm in Prosthetic Joint Infections?**

*Per row, choose one answer* | Rows will be randomized

|  | **Agree** | **Disagree** |
| --- | --- | --- |
| Biofilms are the main cause of delayed onset infection and recurrent infection post-surgery. | **○** | **○** |
| Current surgical site infection diagnosis (using cultures) does not determine if an infection is acute or related to mature biofilm. | **○** | **○** |
| Many biofilm-related infections can return culture negative results. | **○** | **○** |
| Biofilms can form within 24 hours on debris, implants and wound tissues. | **○** | **○** |
| Once mature, biofilms can become resistant to most antibiotic and antiseptic treatments. | **○** | **○** |
| The host immune response against biofilms is limited. | **○** | **○** |
| The presence of biofilm reduces osteoblast viability and differentiation. | **○** | **○** |
| Prevention of biofilm formation by removing debris and killing bacteria before they attach to a surface is a goal of the surgical team. | **○** | **○** |
| Infections caused by antibiotic resistant bacteria pose an increasing challenge in post-surgical complications. | **○** | **○** |
| An irrigation solution that has continued antiseptic activity after closure helps prevent biofilm formation. | **○** | **○** |
| An irrigation solution that has an agent to prevent biofilm adherence to an implant is desirable. | **○** | **○** |

**Q17) Do you agree or disagree with the following statements regarding intraoperative wound irrigation interventions?**

*Per row, choose one answer* | Rows will be randomized

|  | **Agree** | **Disagree** |
| --- | --- | --- |
| Keeping the operative site/ wound free from contaminants is critical to minimize surgical site infection risk. | **○** | **○** |
| Local antiseptic use can complement systemic antibiotic regimen to minimize surgical site infection which supports antimicrobial stewardship. | **○** | **○** |
| Interoperative wound irrigation with saline does not provide sufficient removal of debris and microorganisms from the wound. | **○** | **○** |
| The ability of a surgical wound irrigation product to be used by both pulsatile lavage and different flow or bulb syringe methods is important to accommodate surgeon preferences in a variety of procedures. | **○** | **○** |
| Antiseptic irrigation solutions that do not require additional wash-out steps are preferred to provide residual antimicrobial activity. | **○** | **○** |
| Intraoperative wound irrigation solutions which are effective in preventing biofilm formation for 24 hours are important to reduce surgical site infections. | **○** | **○** |
| Antiseptic irrigants should be safe and not harm implant/prosthetic materials in-situ. | **○** | **○** |
| Antiseptic irrigants should be safe and not harm osteoblasts/osteocytes and cementless bone ingrowth activity. | **○** | **○** |
| Different interoperative wound irrigation protocols should be developed for prevention of surgical site infections versus management of established infections. | **○** | **○** |
| Management and removal of bioburden locally with effective antiseptic irrigants may reduce the incidence of surgical site infections. | **○** | **○** |
| In primary joint replacements, effective antiseptic irrigants are preferred to the use of antibiotic methods in irrigation or cement to reduce antimicrobial resistance and support antimicrobial stewardship. | **○** | **○** |
| In primary joint replacements antiseptic irrigant efficacy post-surgery is preferred over the use of antibiotics within cement. | **○** | **○** |

**Q18) Do you agree or disagree with the following statement regarding in vitro expectations of intraoperative wound irrigation (IOWI)?**

***Per row, choose one answer* | Rows will be randomized**

|  | **Agree** | **Disagree** |
| --- | --- | --- |
| Intraoperative wound irrigation (IOWI) should not compromise cell viability (non-cytotoxic). | **○** | **○** |
| IOWI should be effective against a broad range of microorganisms commonly implicated in surgical site infections. | **○** | **○** |
| IOWI should be able to disrupt biofilm structures. | **○** | **○** |
| IOWI should be effective against antibiotic resistant bacterial strains. | **○** | **○** |
| IOWI that demonstrate strong antimicrobial and antibiofilm properties in clinically relevant in vitro tests may be effective clinically as part of a surgical site infection prevention bundle. | **○** | **○** |
| If an irrigation solution requires a step to completely rinse out with saline following use, this then may reduce effectiveness of antiseptic or antibacterial properties from the original solution. | **○** | **○** |
| Intraoperative contamination can occur during the closure part of the procedure. | **○** | **○** |
| A no rinse irrigation solution with continued efficacy during the post-operative period is preferred to a solution that needs to be rinsed out. | **○** | **○** |

**End of survey questions**
